# Glucuronoyl Esterase of Pathogenic *Phanerochaete carnosa* Induces Immune Responses in Aspen Independently of Its Enzymatic Activity

**DOI:** 10.1111/pbi.70357

**Published:** 2025-09-17

**Authors:** Evgeniy N. Donev, Marta Derba‐Maceluch, Xiao‐Kun Liu, Henri Colyn Bwanika, Izabela Dobrowolska, Mohit Thapa, Joanna Leśniewska, Jan Šimura, Alex Yi‐Lin Tsai, Konrad S. Krajewski, Dan Boström, Leszek A. Kleczkowski, Maria E. Eriksson, Karin Ljung, Emma R. Master, Ewa J. Mellerowicz

**Affiliations:** ^1^ Umeå Plant Science Centre (UPSC), Department of Forest Genetics and Plant Physiology Swedish University of Agricultural Sciences Umeå Sweden; ^2^ Department of Cell and Systems Biology University of Toronto Toronto Ontario Canada; ^3^ Department of Cytophysiology, Faculty of Biology and Environmental Protection University of Lódz Lódz Poland; ^4^ Department of Applied Physics and Electronics Umeå University Umeå Sweden; ^5^ Umeå Plant Science Centre (UPSC), Department of Plant Physiology Umeå University Umeå Sweden; ^6^ Department of Chemical Engineering & Applied Chemistry University of Toronto Toronto Ontario Canada

**Keywords:** biotic stress, glucuronoyl esterase, lignocellulose improvement, *Populus*, PTI, transgenic crops, unfolded protein response

## Abstract

Microbial enzymes expressed in plants add new functionalities but occasionally trigger undesirable immune responses. *Phanerochaete carnosa* glucuronoyl esterase (*Pc*GCE) hydrolyses the bond between lignin and 4‐*O*‐methyl‐α‐D‐glucuronic acid substituent of glucuronoxylan. *Pc*GCE constitutively expressed in *Arabidopsis* or hybrid aspen ( *Populus tremula* × *tremuloides*) improved saccharification but also induced premature leaf senescence. To understand what triggered this senescence, we characterised *Pc*GCE‐expressing hybrid aspen by microscopy and omics approaches, supplemented by grafting and recombinant protein application experiments. *Pc*GCE induced massive immune responses followed by senescence in the leaves. Expressing an inactive (*Pc*GCE^S217A^) enzyme has led to similar phenotypes, excluding a possibility that damage‐associated molecular patterns (DAMPs) released by glucuronoyl esterase triggered immune responses. Grafting experiments showed that *Pc*GCE transcripts are not mobile but they induce systemic responses. Recombinant *Pc*GCE protein applied to leaves did not induce such responses; thus, *Pc*GCE is probably not perceived as a pathogen‐associated molecular pattern (PAMP). We suggest that the observed high expression of *PcGCE* from the 35S promoter triggers the unfolded protein response. Indeed, restricting *PcGCE* expression to short‐lived xylem cells by using the wood‐specific promoter avoided all detrimental effects. Thus, wood‐specific expression is a viable strategy for *PcGCE* deployment *in planta*, which might be applicable for other stress‐inducing proteins.

## 1 Introduction

Saprophytic and pathogenic microbes living on plants developed specific ways of decomposing lignocellulose, and they are a source of enzymes widely used for lignocellulose processing (Thapa et al. 2020). Many of these enzymes and specialised lignocellulose‐interacting proteins are not found in plants but can be introduced to different crops for post‐synthetic modification of their cell walls (Brandon and Scheller 2020) and there are several examples of successful deployment of such proteins *in planta*. For instance, the ferulic acid esterase from *Aspergillus niger* van Thieghem (*An*FAE) introduced to a forage crop tall fescue ( *Festuca arundinacea* Schreb.) increased biomass digestibility by cellulases (de Buanafina et al. 2008, 2010). Fungal acetyl xylan esterases from *A. niger* (*An*AXE1) or from *Hypocrea jecorina* Berkeley & Broome (formerly *Trichoderma reesei* Simmons) (*HjAXE*) expressed in hybrid aspen ( *Populus tremula* L. *x tremuloides* Michx.) increased glucose yield by 20 to 30% in enzymatic saccharification without pretreatment and improved lignin solubility (Pawar et al. 2017) and cell wall nanoporosity (Wang et al. 2020). Even greater saccharification benefits were reported in poplar ( *Populus alba* L.) expressing fungal xylanase *HvXYL1* (Kaida et al. 2009) or in different plant species expressing enzymes targeting xyloglucan and pectins (Park et al. 2004; Kaida et al. 2009; Tomassetti et al. 2015).

However, microbial enzymes expressed *in planta* sometimes trigger immune responses, such as leaf senescence, necrotic lesions in the leaves, accumulation of reactive oxygen species (ROS) and induction of genes involved in biotic and abiotic stress responses (Bailey et al. 1990; Avni et al. 1994; de Buanafina et al. 2012; Pogorelko et al. 2013; Tsai et al. 2017). It has been suggested that their activities could generate oligosaccharides, such as oligogalacturonides or pectin fragments (Legendre et al. 1993; Norman et al. 1999; D'Ovidio et al. 2004; Molina et al. 2021), cellooligomers (Souza et al. 2017), xylogluco‐oligosaccharides (Claverie et al. 2018) or xylo‐oligosaccharides (Mélida et al. 2020; Dewangan et al. 2023), which are perceived as damage‐associated molecular patterns (DAMPs) activating defence responses.

Alternatively, these microbial proteins could be recognised by plants as microbe‐ or pathogen‐associated molecular patterns (MAMPs/PAMPs), as is known for the 22‐amino‐acid N‐terminal peptide of flagellin (flg22) of gram‐negative bacteria or the fragments of chitin present in cell walls of fungal pathogens (Felix et al. 1998, 1999; Latgé 2007; Boller and Felix 2009; Böhm, Albert, Fan, et al. 2014; Raaymakers and Van den Ackerveken 2016). Among the microbial enzymes perceived as PAMPs, the ethylene‐inducing xylanase (EIX) from *Trichoderma viride* Pers. has a conserved peptide motif on the surface of the protein shared with many other microbial xylanases, which is responsible for the binding with the EIX receptor and inducing defence responses in plants independently of the xylanase activity of these enzymes (Bailey et al. 1990; Avni et al. 1994; Enkerli et al. 1999; Rotblat et al. 2002; Ron and Avni 2004; Noda et al. 2010; Frías et al. 2019; Sussholz et al. 2020). Similarly, a microbial GH12 xyloglucanase XEG1 and related microbial GH12 enzymes are recognised as PAMP in soybean ( *Glycine max* ) and solanaceous species (Ma et al. 2015; Wang et al. 2018), fungal polygalacturonases are perceived as PAMPs in *A. thaliana* (Zhang et al. 2014) and *Acremonium strictum* subtilisin (AsES) in strawberry (Caro et al. 2020).

PAMPs and DAMPs are recognised by cell surface‐localised pattern recognition receptors (PRRs) that activate the basal resistance response against a broad range of pathogens, inducing pattern‐triggered immunity (PTI) (Couto and Zipfel 2016; Yu et al. 2017; He et al. 2018). To date, over 60 PRRs for different MAMPs/PAMPs/DAMPs have been characterised in plants (Ngou et al. 2022). Moreover, virulence factors could activate cytoplasmic resistance (R) proteins and initiate effector‐triggered immunity (ETI) (Jones and Dangl 2006; Cui et al. 2015), which amplifies the basal PTI transcriptional programme and triggers localised programmed cell death (PCD) (Balint‐Kurti 2019). Thus, the expression of microbial enzymes in plants potentially could activate defence responses, immunity and even cell death. Indeed, transgenic plants expressing microbial enzymes frequently exhibit increased immunity against different pathogens (Pogorelko et al. 2013; Klose et al. 2015; Pawar et al. 2016; Reem et al. 2020) although the molecular mechanisms behind these reactions are far from being understood.

Among different microbial enzymes, glucuronoyl esterase (GCE) has a potential for decreasing cross‐linking of cell wall polymers by hydrolysing the ester bond between 4‐*O*‐methyl‐α‐D‐glucuronic acid and lignin (Spániková and Biely 2006; Biely et al. 2015; Arnling Bååth et al. 2016). This bond is thought to mediate the formation of lignin‐carbohydrate complexes (LCCs) in woody species, considered crucial for lignocellulose recalcitrance (Giummarella et al. 2019). To reduce this recalcitrance, a GCE from necrotrophic softwood decaying white‐rot basidiomycete, *Phanerochaete carnosa* Burt (*Pc*GCE) was ectopically expressed in *Arabidopsis thaliana* (L.) Heynh. and hybrid aspen, resulting in reduced cell wall cross‐linking (Tsai et al. 2012) and increased cellulose‐to‐glucose conversion despite the highly elevated lignin content (Gandla et al. 2015), respectively. However, both species exhibited premature leaf senescence and growth retardation, indicating that additional work is required to understand *Pc*GCE effects *in planta* and to design a better strategy to express it for saccharification improvement. To elucidate the cause of untargeted effects of *Pc*GCE in *A. thaliana* , Tsai et al. (2017) characterised transcriptomic responses to the ectopically expressed enzyme. They identified massive transcriptome reprogramming but were not able to assign whether PAMP‐, DAMP‐ or other pathway was involved in these responses. Here we analysed responses to *Pc*GCE in hybrid aspen in more detail, determining changes in leaf anatomy, transcriptomes, metabolomes, levels of their hormones and reactive oxygen species (ROS). We followed the progress of defence responses during leaf development and their transmission through grafts. We also studied effects of applied native and mutated, enzymatically inactive *Pc*GCE protein and effects of expressing the mutated *Pc*GCE in aspen. The results indicated that *Pc*GCE is a potent inducer of stress responses regardless of its enzymatic activity and that it is likely not perceived as a PAMP. Moreover, all undesirable responses to *Pc*GCE could be avoided by using the wood‐specific promoter, providing a strategy for its deployment in transgenic crops dedicated to biorefinery.

## 2 Results

### 2.1 Ectopically Expressed *Pc*GCE Induced Developmental Defects and Defence Responses in Aspen

**FIGURE 1:**
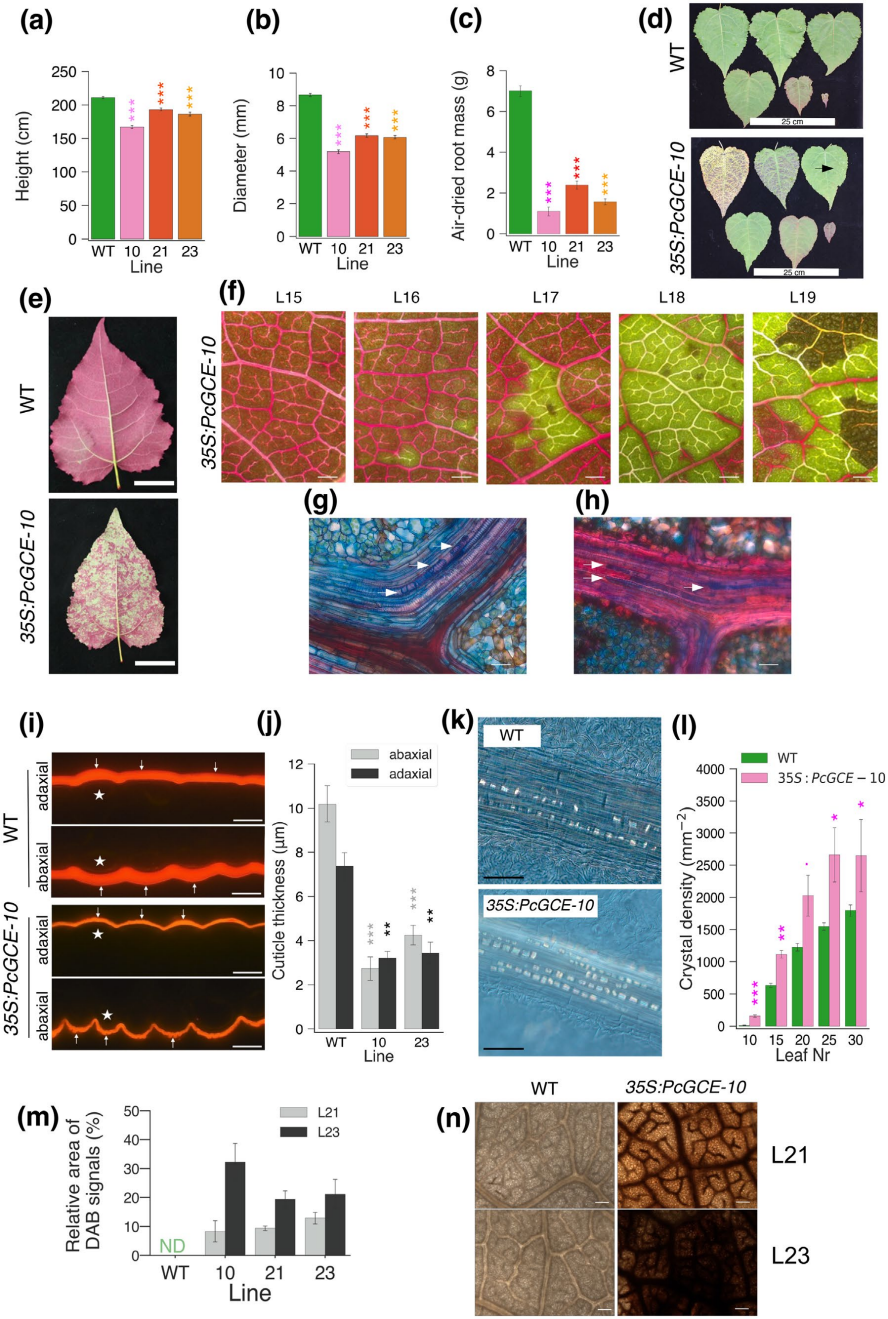
Transgenic aspen constitutively expressing *PcCGE* display different disease symptoms. Height (a), diameter (b) and root weight (c) of 11‐week‐old plants of three independent transgenic lines: *35S:PcGCE*‐10, ‐21 and ‐23 and WT. (d) Appearance of leaves 5, 10, 15, 20, 25 and 30 of *35S:PcGCE*‐10 and WT plants. Note the rapid progression of necrosis in transgenic leaves from the first occurrence (arrow). (e, f) Hydraulic continuity is compromised in transgenic leaves as shown by the dye uptake inhibition. Green areas indicate hydraulic blockage. (e) Appearance of WT and transgenic (*35S:PcGCE*‐10) leaves after dye uptake. Scale bar = 5 cm. (f) Progression of dye uptake blockage and necroses in successive leaves (L15‐L19) of a transgenic plant (*35S:PcGCE*‐10). Scale bar = 500 μm. (g, h) Blockage of xylem vessels by tyloses (g, arrows) and gels (h, arrows) in the leaves of transgenic plants (*35S:PcGCE*‐10). Longitudinal sections through vascular bundles stained with safranin and alcian blue. Scale bars = 20 μm. (i) Cuticle (arrows) in leaf epidermis of Line 10 and WT plants stained with Nile red. * is placed over an epidermal cell, scale bars: 10 μm. (j) Cuticle thickness in transgenic lines and WT. (k) Calcium oxalate crystals along the leaf veins of line *35S:PcGCE*‐10 and WT. Note the higher density and larger size of crystals in transgenic plants. Scale bar = 100 μm. (l) Crystal density over veins in leaves 10–30 of line *35S:PcGCE*‐10 and WT. (m, n) *Pc*GCE induces ROS accumulation. Symptomless leaves (L21) and leaves with first necroses (L23) analysed by diaminobenzidine (DAB) staining. (m) Area of DAB signals quantified by image analysis. ND—not detected. (n) Light microscopy images of representative leaves of line *35S:PcGCE*‐10 and WT. Scale bar = 100 μm. (a, b, c, j, l, m) Means ± SE, *N* = 24 for transgenic lines and 40 for WT in (a, b), 6 in (c), 3 in (j), 12 in (l, m). Asterisks show significant differences compared to WT at: ^.^ *p* < 0.1; *p ≤ 0.05; **p ≤ 0.01;***p ≤ 0.001 (Dunnet's or *t*‐test).

Hybrid aspen lines ectopically expressing *Pc*GCE displayed reduced growth, leaf necrosis and premature shedding (Figure 1a–d; Gandla et al. 2015). The necrotic spots first appeared on fully expanded leaves and they rapidly enlarged, occupying most of the leaf area, followed by premature shedding. Using a dye uptake analysis, we found that the hydraulic continuity was compromised in the leaves of transgenic plants before the necrotic spots appeared. This was visible as unstained areas on leaves of branches immersed in a staining solution (Figure 1e,f). Anatomical analyses revealed that xylem vessels were blocked by gels and tyloses (Figure 1g,h), indicative of the activation of defence responses to pathogen attack and/or herbivory and signalling by ethylene (ET) and jasmonic acid (JA) (Leśniewska et al. 2017). We further found that the thickness of the cuticle on the abaxial and adaxial epidermis of transgenic plants was strongly reduced (Figure 1i,j). Moreover, the prismatic crystals localised mostly along the veins displayed higher density and larger size in transgenic plants compared to wild‐type (WT) plants (Figure 1k,l; Figure S2). The chemical identity of the crystals was established by x‐ray diffraction as calcium oxalate (CaOx) or whewellite, CaC_2_O_4_ × H_2_O, which is known to be induced by herbivory (Molano‐Flores 2001). The transgenic plants were also found to display highly elevated ROS levels in fully expanded leaves (L21 and L23) based on diaminobenzidine (DAB) staining (Figure 1m,n). Thus, the ectopic *Pc*GCE expression induced a variety of defence responses in aspen leaves, culminating in leaf necrosis and premature shedding.

### 2.2 Molecular Changes Caused by *Pc*GCE in Aspen Leaves

**FIGURE 2:**
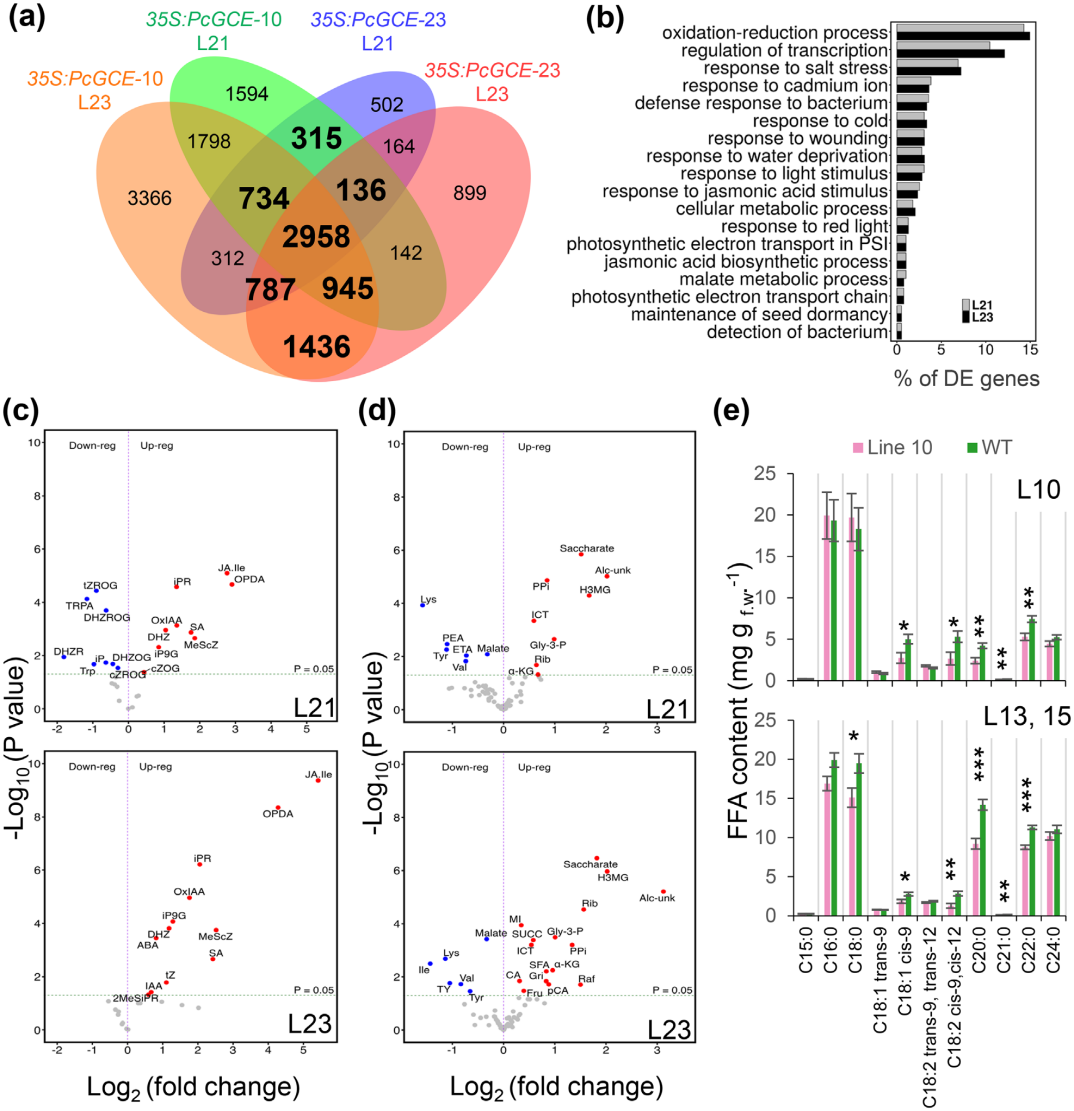
Overview of transcriptomic and metabolomic changes in aspen ectopically expressing *PcGCE*, compared to wild‐type. (a‐d) Symptomless leaves (L21) and leaves with necrotic spots (L23) were analysed in lines *35S:PcGCE*‐10, ‐23 and WT. (a) Venn diagram of differentially expressed (DE) genes. (b) Gene Ontology (GO) enrichment analysis of DE genes in both transgenic lines. (c, d) Volcano plots showing altered levels of metabolites detected by hormonomics (c) and metabolomics (d) analyses. The significantly altered metabolites compared to WT are labelled. Abbreviations (c): ABA—abscisic acid; cZOG—cis‐zeatin‐*O*‐glucoside; cZROG—cis‐zeatin riboside‐*O*‐glucoside; DHZ—dihydrozeatin; DHZR—dihydrozeatin riboside; DHZOG—dihydrozeatin‐*O*‐glucoside; DHZROG—dihydrozeatin riboside‐*O*‐glucoside; iP—N6‐isopentenyl‐adenine; iPR—N6‐iso‐pentenyl‐adenosine; JA—jasmonic acid; JA‐Ile—isoleucine‐JA; MeScZ—2‐methylthio‐*cis*‐zeatin; MeSiPR—2‐methylthio‐isopentenyladenosine; IAA—indole‐3‐acetic acid; iP9G—N6‐isopentenyladenine‐9‐glucoside; OPDA—*cis*‐12‐oxo‐phytodienoic acid; OxIAA—2‐oxoindole‐3‐acetic acid; SA—salicylic acid; Trp—tryptophan; TRPA—tryptamine; tZ—trans‐zeatin; tZROG—trans‐zeatin‐riboside‐*O*‐glucoside. (d): Alc‐unk—unknown alcohol; CA—caffeate; ETA—EtOH‐amine; Fru—fructose; Gly‐3‐P—glycerol‐3‐P; Gri—glyceric acid; H3MG—hydroxy‐3‐methylglutaric acid; ICT—iso‐citrate; Ile—iso‐leucine; α‐KG—keto‐glutarate; Lys—lysine; MI—*myo*‐inositol; pCA—*p*‐coumarate; PEA—phenetyl‐amine; PPi—pyro‐phosphate; Rib—ribose; SUCC—succinate; SFA—sulphamic acid; TY—tyramine; Val—valine. (e) Free fatty acid (FFA) contents in expanding (L10) and expanded (L13 and L15) leaves of transgenic (*35S:PcGCE*‐10) and WT plants. Data are means ± SE of at least four biological replicates per line. Asterisks show significant differences between transgenic and WT plants (ANOVA Fisher's test, *p* ≤ 0.05 *; *p* ≤ 0.01 **; *p* ≤ 0.001 ***).

To characterise the molecular pathways involved in these defence responses, we analysed transcriptomes, metabolomes and hormonomes in fully expanded leaves (L21 and L23) of two transgenic lines, *35S:PcGCE‐10* and *35S:PcGCE‐23*. *Pc*GCE massively disrupted transcriptome profiles of leaves with as many as 16,087 genes (39% of all protein‐coding genes) differentially expressed (DE) in at least one line and at least in one leaf developmental stage (Table S1), and 4143 and 6126 genes (10 and 15% of protein coding genes) differentially expressed in L21 and L23, respectively, in both transgenic lines (Figure 2a; Table S2). The gene ontology (GO) analysis of DE genes in both lines (Figure 2b) revealed enrichment in oxidation–reduction processes, regulation of transcription, defence signalling in biotic and abiotic stresses, signalling by JA, malate metabolism and photosynthesis. Among redox‐related genes, there were 21 peroxidases, which were all upregulated, indicating increased ROS levels (Table S2). The majority of 517 DE transcription factors (TFs) were downregulated. Opposite to this general trend, several MYB (similar to *At*MYB15, 62, 63, 66, 68, 73, 84 and 116) and WRKY (similar to *At*WRKY6, 51 and 75) TFs were highly upregulated. The biotic stress‐related DE genes (175 genes) were mostly upregulated, whereas abiotic stress response genes (149 genes) were mostly downregulated. Almost all JA‐related DE genes (51) were upregulated, whereas the majority of SA‐related DE genes (18) were downregulated. ET‐related DE genes (105) were strongly either up‐ or downregulated. Among 118 photosynthesis‐related DE genes, the majority were downregulated.

The hormone profiling (Šimura et al. 2018) provided evidence of hormonal signalling downstream of *Pc*GCE. There was a strong induction of JA and its precursor 12‐oxophytodienoic acid (OPDA) and an increase in SA, which was evident already in symptomless leaves (L21) and became more prominent in older necrotic leaves (L23) (Figure 2c). Several cytokinin‐ and few auxin‐related metabolites were altered, and ABA increased in older leaves.

Metabolomic analysis (Figure 2d) revealed upregulation of hydroxy‐3‐methyl glutaric acid (mevalonate), *myo*‐inositol, glycerol‐3‐phosphate (Gly‐3‐P), saccharate, ribose and metabolites related to the TCA cycle, such as iso‐citrate and keto‐glutarate. In contrast, the contents of amino acids (Ile, Lys, Tyr, Val) and malate were reduced. Contents of fructose, raffinose, succinate, *p*‐coumaric acid and caffeic acid were upregulated in older leaves. Mevalonate (Nelson et al. 1994) and Gly‐3‐P (Chanda et al. 2008) have been associated with basal resistance and induction of systemic immunity. Also, enhanced levels of sugars have been observed during defence responses (Gómez‐Ariza et al. 2007; Essmann et al. 2008; Conrath 2011). Lower amino acid content corresponds to a general decrease in protein biosynthesis and amino acid metabolism reflected in the transcriptome (Table S2).

As the lipid metabolism is known to be affected during stress responses (Kachroo and Kachroo 2009), we analysed early changes in free fatty acids (FFAs) in expanding (L10) and expanded (L13 and L15) leaves of line 10 and WT. As the principal component analysis (PCA) revealed differences in FA profiles of expanding and expanded leaves (Figure S1), we analysed these groups separately. While the FA profiles differed between these groups, both groups exhibited decreased levels of oleic acid (C18:1 *cis*‐9), linoleic acid (C18:2 *cis*‐9, *cis*‐12) and very long chain FAs (VLCFA) in transgenic plants compared to WT (Figure 2e), which could reflect a diversion of lipid metabolism to JA biosynthesis.

Overall, the omics analyses revealed heavily disrupted transcript and metabolite profiles in transgenic plants, with activation of general stress responses and downregulation of growth and development.

### 2.3 Sequential Analysis of Developing Leaves Revealed Three Stages of Defence Responses Development

**FIGURE 3:**
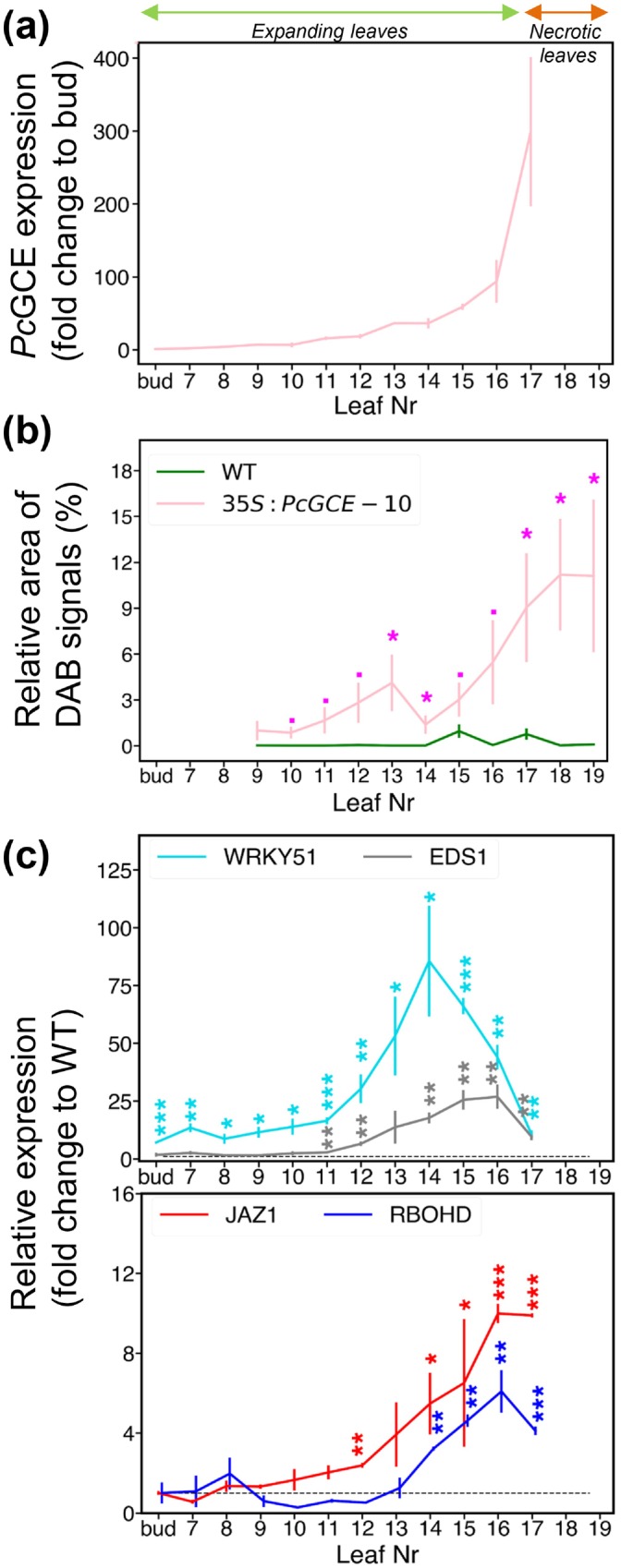
Expression of *PcGCE* and development of defence reactions in sequential leaves of 8‐week‐old transgenic plants of line *35S:PcGCE*‐10. (a) *PcGCE* transcript levels analysed by the reverse transcription–quantitative polymerase chain reaction (RT‐qPCR). (b) ROS levels analysed by DAB staining. (c) Transcript levels of defence response marker genes: *WRKY51* (*Potri.005G085200*), *EDS1* (*Potri.015G069600*), *JAZ1 (Potri.003G068900*) and *RBOHD* (*Potri.001G070900*), determined by RT‐qPCR, relative to WT (stiped line). Data are means ± SE, *N* = 3 in (a, c) or 12 in (b). Asterisks show significant differences compared to WT (*t*‐test; ^.^ *p* < 0.1; *p ≤ 0.05; **p ≤ 0.01; ***p ≤ 0.001).

To understand the progress of defence responses in developing transgenic leaves, we analysed the developmental leaf series covering the entire leaf expansion phase in line *35S:PcGCE‐10* until the first necrotic spot appeared (L17). *Pc*GCE expression analysed by reverse transcription quantitative polymerase chain reaction (RT‐qPCR) showed that *Pc*GCE mRNA accumulated exponentially in developing leaves with the acceleration from leaf 14 (Figure 3a). ROS, analysed by DAB staining in sequential leaf samples from the first unfolded leaf (L9) until necrotic spots developed (L17‐ L19) in line *35S:PcGCE‐10* and in the corresponding series in WT, showed no signals in WT whereas transgenic leaves had a small peak in half‐expanded leaves (L13) and a large peak associated with the development of necrotic spots (L18) (Figure 3b). *Potri.005G085200*, a homologue of *Arabidopsis WRKY51* known to be induced by low levels of oleic acid (Gao et al. 2011), was highly upregulated by *Pc*GCE from the earliest leaf developmental stages, peaking in L14 and then decreasing (Figure 3c). A homologue of *EDS1* involved in the hypersensitive response (HR) and antagonising JA signalling (Gao et al. 2011) (*Potri.015G069600*) and a homologue of *JAZ1* representing the JA signalling pathway responsive to JA levels (Chung et al. 2008) (*Potri.003G068900*) were upregulated in transgenic plants starting from L11 and L12, respectively. *Potri.001G070900*, a homologue of *RBOHD* representing the ROS generating pathway of systemic acquired resistance (SAR) triggering HR response (Torres et al. 2002; Nühse et al. 2007; Lew et al. 2020), was upregulated at later developmental stages (L14‐L17) (Figure 3c). Thus, the analysis revealed three main stages in the development of defence responses in the leaves of transgenic plants, namely an early stage dominated by *WRKY51* (bud‐L9), a middle stage with activation of JA signalling and *EDS1* expression accompanied by a small peak in ROS (L10‐L14) and a late stage characterised by high ROS accumulation and HR (L16‐L19).

### 2.4 Grafting Experiments Revealed That *PcGCE* mRNA Is Not Cell‐To‐Cell Mobile but Defence Responses Are Induced at a Distance by *Pc*GCE

**FIGURE 4:**
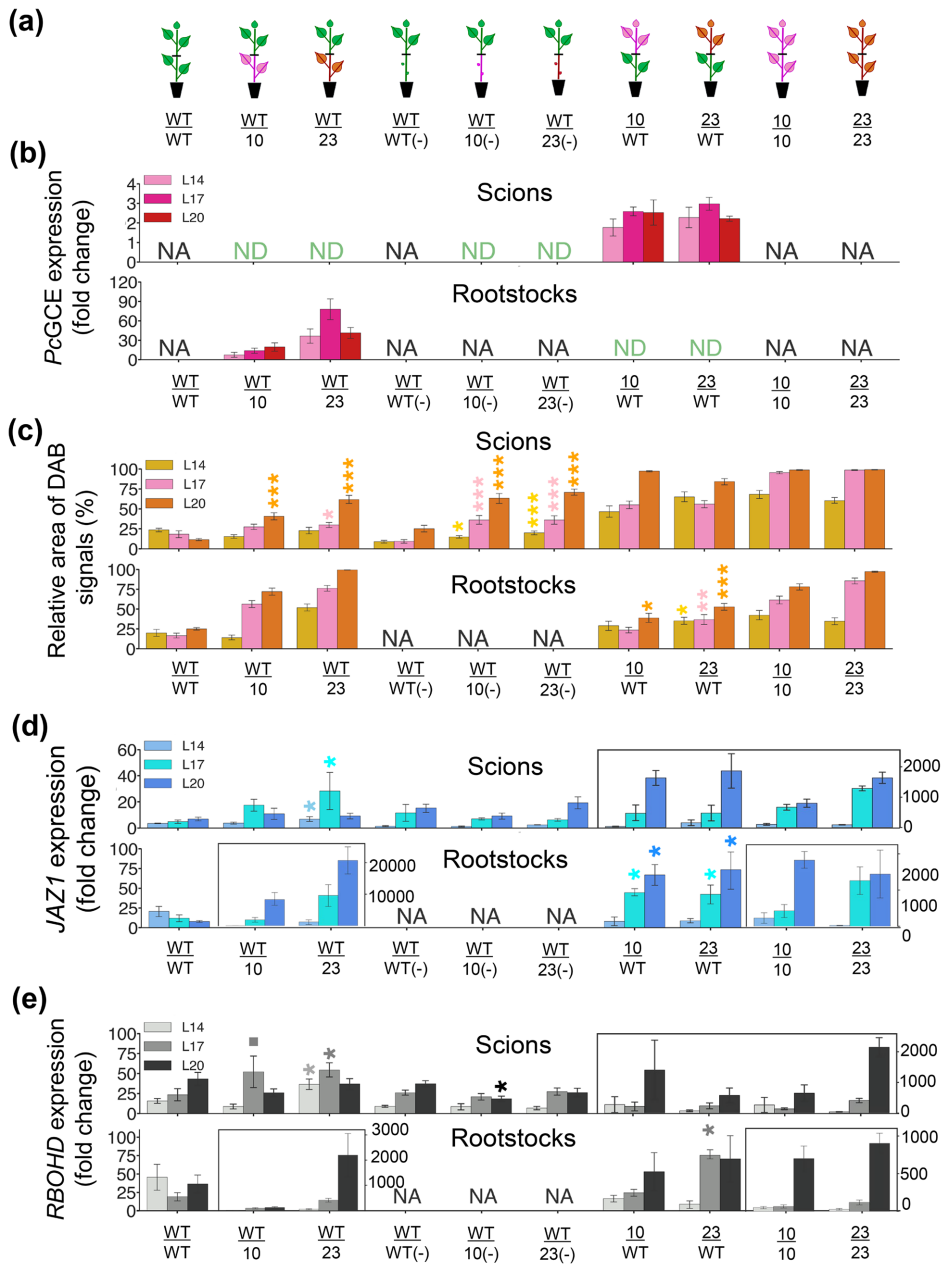
Grafting experiments indicate that *PcGCE* transcripts are not cell‐to‐cell mobile and that defence responses are induced by *Pc*GCE at a distance. (a) Schematics of different types of grafts between WT and transgenic plants of lines *35S:PcGCE*‐10 and *35S:PcGCE*‐23. (−) indicates rootstocks with removed leaves. (b–e) *PcGCE* transcript levels determined by RT‐qPCR (b), ROS contents determined by DAB staining and image analysis (c), marker gene expression analysed by RT‐qPCR (d, e). Leaves L14, L17 and L20 of scions and rootstocks were analysed 48 days after grafting. Analyses in rootstocks were performed on side shoots to obtain similar leaf developmental stages as in scions. ND—not detected; NA—not analysed. Expression values in (b, d, e) are fold changes relative to the least expressing sample. Asterisks show significant differences compared to WT/WT or WT/WT(−), as appropriate (*t*‐test, *p* ≤ 0.1; *p ≤ 0.05; **p ≤ 0.01; ***p ≤ 0.001). Other comparisons are not shown.

To distinguish between local and systemic responses to *Pc*GCE, the scions of 5‐week‐old transgenic and WT plants were grafted onto the transgenic or WT rootstocks in all possible combinations (Figure 4a). *Pc*GCE transcripts were found only in the transgenic leaves of scions and rootstocks (Figure 4b), indicating that they were not cell‐to‐cell mobile. In contrast, induction of ROS and *JAZ1* and *RBOHD* expression was observed in leaves of WT scions grafted on transgenic rootstocks and WT rootstocks carrying transgenic scions (Figure 4c,d), indicating that these defence responses are graft‐transmittable and induced at a distance by *Pc*GCE. Furthermore, the removal of leaves from transgenic rootstocks (Figure 4) had a positive impact on the systemic ROS induction in WT leaves of scions when compared with WT leaves of scions grafted on WT leafless rootstocks (Figure 4c). In contrast, the removal of leaves from transgenic rootstocks completely prevented *JAZ1* and *RBOHD* induction in WT leaves of scions (Figure 4d), pointing to differences in ROS propagation and systemic gene induction pathways.

### 2.5 Expressing of *PcGCE* From Wood‐Specific Promoter Allows Normal Growth

**FIGURE 5:**
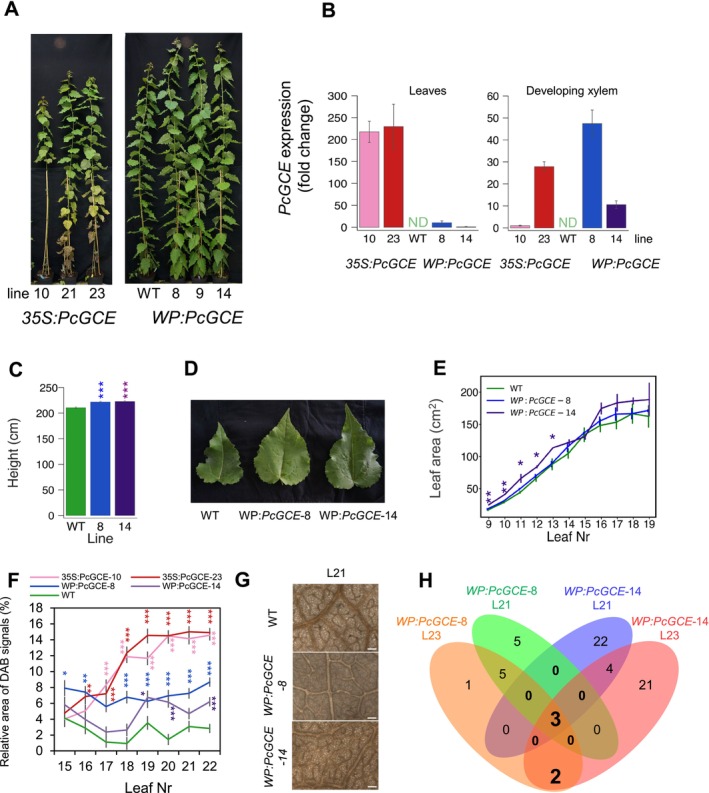
Transgenic aspen expressing *PcGCE* from the *WP* promoter does not show disease symptoms during the 11‐week cultivation in the greenhouse. (a) Appearance of *WP:PcGCE*‐8, ‐9 and ‐14 lines compared to WT and 35S:*PcGCE* lines. (b) Transgene expression in mature leaves (L21) and developing xylem analysed by RT‐qPCR normalised to the lowest expressing line. No expression was detected in WT plants (ND). Height (c), appearance of leaves (L19) (d) and leaf area (e) of 11‐week‐old plants. (f) ROS levels in leaves analysed by DAB staining and image analysis in WP:*Pc*GCE lines compared to 35S:*Pc*GCE lines and WT. (g) Representative images of DAB‐stained leaf 21 in *WP:PcGCE* and WT plants. Scale bar = 100 μm. Data in (b, c, e, f) are means ± SE. *N* = 3 (b), 12–20 (c, e), or 24 (f). Means significantly different from WT are shown with asterisks (Dunnett's test, *p ≤ 0.05; **p ≤ 0.01; ***p ≤ 0.001). (h) Venn diagram of differentially expressed genes in mature leaves (L21, L23) of transgenic lines: *WP:PcGCE*‐8 and ‐14 compared to WT.

Because *PcGCE* transcripts are not mobile, expressing *Pc*GCE in a tissue that is not affected by this enzyme should avoid the development of stress symptoms. We hypothesised that developing wood might be such a tissue. To test it, we expressed the transgene using the wood‐specific promoter (WP) that is activated in cells depositing secondary walls (Ratke et al. 2015). The three most highly expressing lines were grown in the greenhouse for 11 weeks, but they did not show any stress symptoms or growth defects, whereas *35S:PcGCE* lines exhibited pronounced premature leaf senescence and necrosis (Figure 5a). The *PcGCE* transcript levels in the leaves were lower in *WP:PcGCE* lines compared to the *35S:PcGCE* lines (Figure 5b), which was expected because there are very few cells developing secondary walls in the leaves. In contrast, in developing wood, the two types of transgenic plants exhibited comparable transgene expression. The stem height and leaf size in *WP:PcGCE* lines were not affected or slightly increased compared to WT (Figure 5c–e) but the ROS levels in the leaves were slightly higher compared to WT (Figure 5f,g). However, in contrast to *35S:PcGCE* lines that exhibited a progressive increase in ROS during leaf development (Figure 3b), the levels of ROS in *WP:PcGCE* lines remained stable (Figure 5f).

Transcriptomic analysis in leaves L21 and L23 revealed only 62 DE genes compared to WT in at least one line and one leaf developmental stage (Figure 5i; Table S3) and only three and five of them were DE in both investigated lines in L21 and L23, respectively. These commonly DE genes included a growth‐related cellulase *PtCel9B3* (Takahashi et al. 2009) which was upregulated, and two downregulated genes: a homologue of *AtPSKR1* encoding a phytosulfokine receptor (Mosher et al. 2013) and *AtMLO5* encoding a Ca^2+^ channel (Meng et al. 2020; Table S3). Hormonomics analysis revealed changes in a few cytokinin‐related species and in tryptamine that were similarly affected as *35S:PcGCE* lines but with much reduced intensity; however, no upregulation of stress‐related hormones was evident (Table S4). Metabolomic analysis showed slight upregulation of some mono‐ and disaccharides (Table S5), which were seen strongly upregulated in *35S:PcGCE* lines, but no changes in metabolites related to the TCA cycle, inorganic phosphate, caffeic acid and amino acids, indicating that the metabolic pathways affected in lines with *35S* and *WP* promoters are not overlapping. These data clearly show that the growth inhibition and stress‐related symptoms that were observed in *35S:PcGCE* expressing plants were avoided when the same protein was expressed from the *WP* promoter.

### 2.6 Transcriptomics of Early Stages of Leaf Development in *35S* : *PcGCE* Plants Identified Putative Receptors and Signalling Components Involved in the Response to *Pc*GCE Protein

**FIGURE 6:**
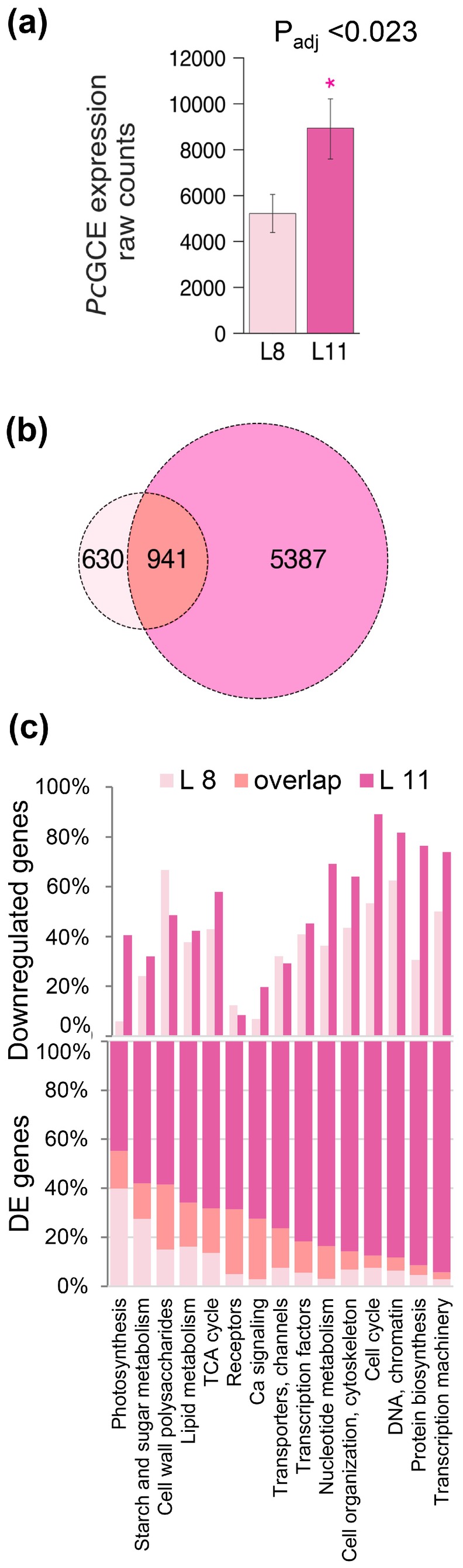
Transcriptomic changes in young aspen leaves (L8 and L11) of line *35S:PcGCE‐10* compared to wild‐type. (a) *PcGCE* transcript levels from raw counts analysis. (b) Venn diagram of differentially expressed (DE) genes in transgenic compared to WT plants. (c) Percentage of DE genes and downregulated genes in selected functional groups. All DE genes and functional enrichment are listed in Tables S6 and S7, respectively. First unfolded leaf (L8) and young expanding leaf (L11) were analysed in five trees.

We analysed transcriptomes in the first unfolded leaf (L8) and in rapidly expanding leaf (L11) in *35S:PcGCE*‐10 and WT plants. This analysis aimed at revealing genes that might be involved in the early stages of signal perception and transduction of *Pc*GCE triggered stress and reducing contribution of genes related to secondary effects evident in the first transcriptomic analysis. Expression of *Pc*GCE was approximately two times higher in L11 than in L8 (Figure 6a), whereas the number of DE genes increased four times from 1571 in L8 to 6328 in L11, with 941 genes overlapping between the two developmental stages (Figure 6b; Table S6). GO enrichment analysis of *Arabidopsis* homologues of DE genes showed 65 significantly affected processes in L8 and 41 largely different processes affected in L11 (Table S7). The house‐keeping activities and development (protein biosynthetic machinery, cell cycle, general transcriptomic machinery, DNA and chromatin organisation and cytoskeleton) were prominent functions affected (largely downregulated) in L11 but they were low represented in L8 (Figure 6c; Table S6). In comparison, proportionally more genes in categories ‘Receptors’ and ‘Ca signalling’ were affected in L8 and these categories tended to be upregulated. Relatively larger proportions of genes uniquely affected in L8 were found for the ‘Photosynthesis’ and ‘Starch and sugars metabolism’. These genes tended to be upregulated. ‘Cell wall polysaccharide’ related genes were by large downregulated in L8 and this trend tended to be reversed in L11. Thus, the responses recorded in transcriptomes at earliest leaf developmental stages of transgenic plants were different from those seen at later stages, and they indicated stimulated biosynthesis of photosynthetic machinery and induction of different proteins involved in Ca signalling. These responses were followed by general downregulation of house‐keeping activities in older leaves, and further development of stress responses.

### 2.7 Inactive *Pc*GCE Induces Similar Stress Responses in Aspen as the Active Enzyme

**FIGURE 7:**
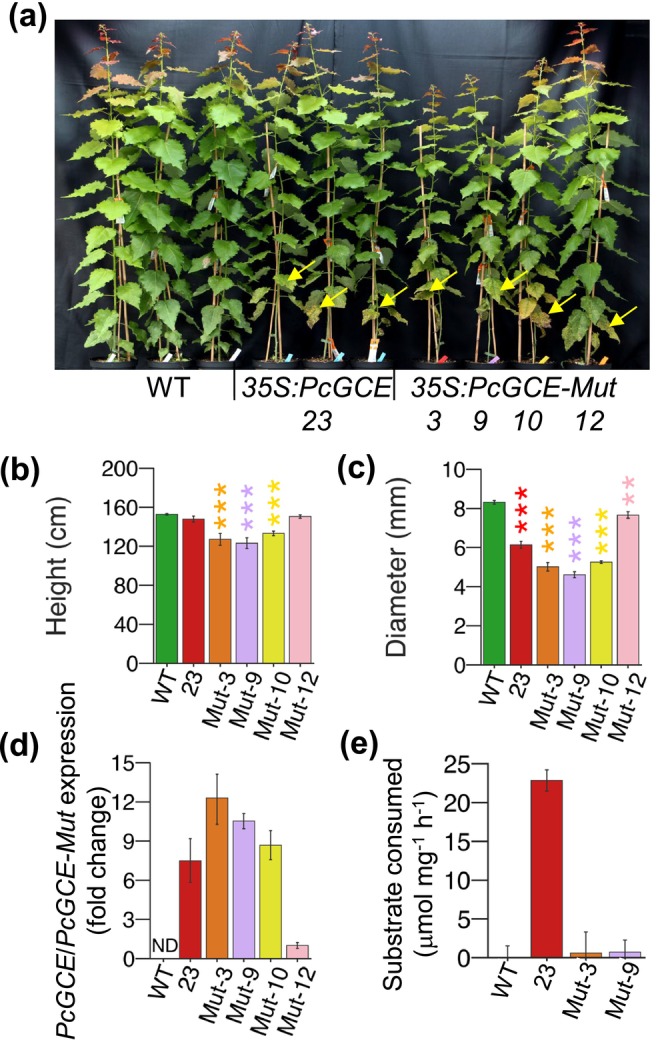
Enzymatic activity of *Pc*GCE is not needed to induce defence responses in aspen. Appearance (a), height (b), diameter (c), transgene transcript levels (d) and glucuronoyl esterase specific activity in mature necrosis‐free leaves (e) of trees expressing mutated, enzymatically inactive *Pc*GCE^S217A^ from the *35S* promoter (*35S:PcGCE‐Mut*) as compared to WT trees and the *35S:PcGCE‐23* line expressing native *Pc*GCE, after the 7‐week cultivation in the greenhouse. Note the premature leaf senescence and necrotic leaves in the *35S:PcGE* and *35S:PcGCE‐Mut* lines (arrows in a). ND‐not detected. Means ± SE, *N* = 7 (b, c), 4 (d, e), asterisks show means significantly different from WT (Dunnett's test; *p ≤ 0.05; **p ≤ 0.01; ***p ≤ 0.001).

It has previously been suggested that *Pc*GCE could be perceived as a PAMP or its enzymatic activity could generate cell wall damage signals perceived as DAMP (Tsai et al. 2017). To further test the DAMP hypothesis, a point mutation S217A was generated in the conserved catalytic triad of glucuronosyl esterases active site (Pokkuluri et al. 2011) of *Pc*GCE, which abolished its glucuronoyl esterase activity (Figure S2). We then expressed the inactive *Pc*GCE^S217A^ in hybrid aspen under the control of the *35S* promoter. All transgenic lines expressing the mutated enzyme displayed leaf necrosis and premature senescence, and the three lines (3, 9 and 10) most highly expressing the transgene had reduced growth after the 7‐week cultivation in the greenhouse (Figure 7a–d). Glucuronoyl esterase activity was detected in leaf extracts of Line 23 expressing wild‐type *Pc*GCE but not in Lines 3 and 9 highly expressing *Pc*GCE^S217A^ (Figure 7e). The data indicate that the glucuronoyl esterase activity is not needed for triggering defence responses *in planta* and therefore we can exclude the DAMP hypothesis.

**FIGURE 8:**
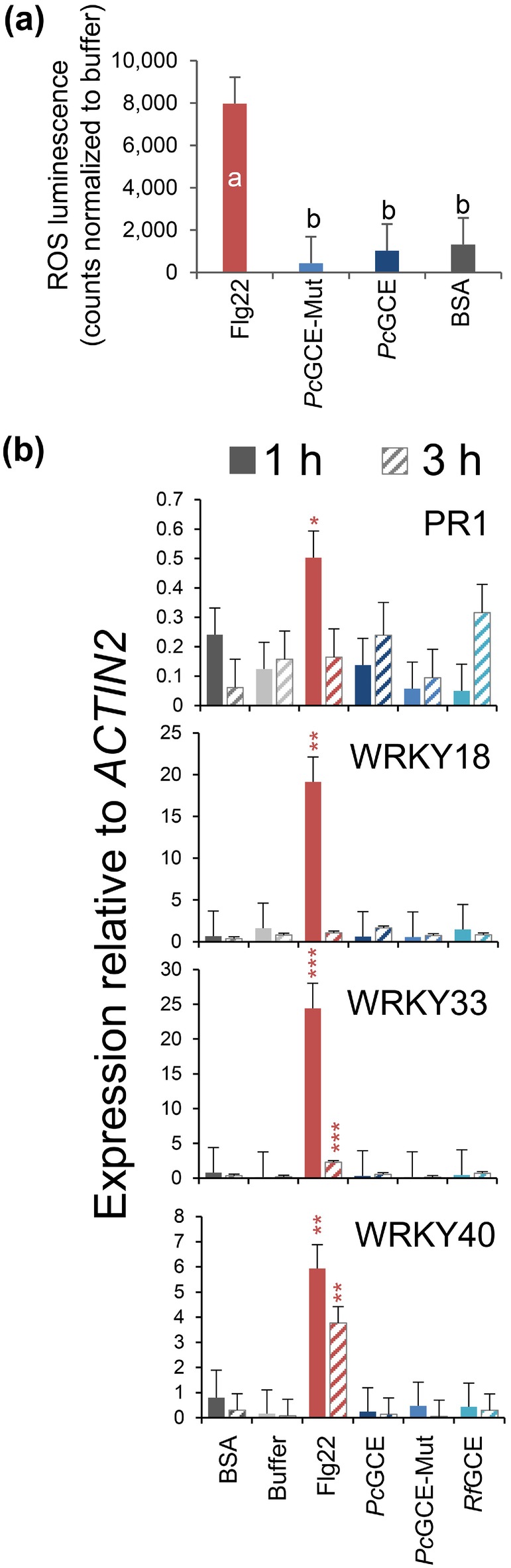
Neither *Pc*GCE nor its inactive form *Pc*GCE^S217A^ (*Pc*GCE‐Mut) induces stress symptoms when applied exogenously. (a) Luminol‐based assay for detection of ROS in aspen leaves treated with different elicitors. (b) Induction of marker genes after treatment of *Arabidopsis* seedlings with different elicitors. Flg22 peptide and bovine serum albumin (BSA) were used as positive and negative controls, respectively. *Rf*GCE— *Ruminococcus flavefaciens* glucuronoyl esterase. Means ± SE, *N* = 5 (a) or 4 (b). Different letters in (a) show significant differences among treatments (Tukey`s test, *p* ≤ 0.05). Asterisks in (b) show means significantly different from buffer (Dunnett's test; *p ≤ 0.05; **p ≤ 0.01; ***p ≤ 0.001).

To corroborate the hypothesis that *Pc*GCE is recognised as a PAMP by a pattern‐recognising receptor (PRR), we designed two pharmacological studies using *Pc*GCE and *Pc*GCE^S217A^ proteins produced in *P. pastoris* to test their elicitor activities. First, we tested if these proteins can rapidly induce ROS in aspen leaves using the luminol assay (Bisceglia et al. 2015). Aspen leaf discs were treated with either *Pc*GCE, *Pc*GCE^S217A^, flagellin‐derived flg22 peptide (positive control) or bovine serum albumin (BSA, negative control). Neither active nor inactive *Pc*GCE induced ROS in aspen leaves above the response to BSA (Figure 8a), indicating these proteins, when exogenously applied to leaves, do not induce ROS as an early response.

We further tested if these proteins could induce marker gene expression when applied to *Arabidopsis* seedlings as it is known for other elicitors perceived by the plasma membrane located PRRs (Böhm, Albert, Fan, et al. 2014; Böhm, Albert, Oome, et al. 2014). The 9‐day‐old *Arabidopsis* seedlings growing on the agar plates were treated with *Pc*GCE, *Pc*GCE^S217A^, a non‐related glucuronosyl esterase (*Rf*GCE), flg22 peptide (positive control) and BSA (negative control) at a 1 μM concentration, and the expression of marker genes for elicitation was tested after 1 and 3 h. The marker genes were selected based on their high induction in transgenic aspen and *Arabidopsis* expressing *Pc*GCE based on our transcriptomics results and the previously published data (Tsai et al. 2017), as listed in Tables S2 and S6. Only flg22 peptide induced the tested marker genes among all tested proteins (Figure 8b). Thus, we did not find support for the hypothesis that *Pc*GCE is recognised as a PAMP.

## 3 Discussion

*P. carnosa* is a basidiomycete pathogen of forest trees found on bark and wood (Burt 1925). As it has the ability to decompose wood causing white rot, it is considered a source of potent lignocellulolytic enzymes for a wide range of technological applications including transgenic expression *in planta* for improvement of saccharification (MacDonald et al. 2011; Suzuki et al. 2012). Glucuronoyl esterase *Pc*GCE is one of such enzymes, but when ectopically expressed in *Arabidopsis* (Tsai et al. 2012) or in aspen (Gandla et al. 2015) with the aim to reduce lignin‐carbohydrate cross‐links in lignocellulose (Arnling Bååth et al. 2016), it induced premature leaf senescence. Much more striking effects of ectopically expressing *Pc*GCE were seen in hybrid aspen. The leaves gradually developed immune defence responses including blockage of xylem by gels and tyloses, accumulation of calcium oxalate crystals, increase of ROS, cell death as evidenced by the necrotic spots and finally premature leaf shedding (Figure 1). Transcriptomics analyses in both *Arabidopsis* (Tsai et al. 2017) and hybrid aspen (Figures 2, 6; Tables S2, S6 and S7) revealed activation of biotic stress and plant defence responses. The transcriptomes of aspen leaves expressing the enzyme were massively altered long before premature senescence was evident. The earliest recorded changes included the induction of *WRKY51* homologue, upregulation of photosynthesis, alteration of sugar and lipid metabolism and activation of signalling involving Ca^+2^, JA and ET (Figures 1, 2, 3 and 6; Tables S6 and S7). These changes were followed by an activation of SAR as evidenced by an increase in ROS content, *RBOHD* and peroxidase expression and SA signalling (Figures 1, 2, 3 and 6; Tables S2 and S6). In parallel, the increases in the levels of JA, SA and ABA and changes in cytokinin's profile were recorded (Figure 2c). These responses are typical for the biotic stress initiated by PTI signalling (Yu et al. 2017; Ngou et al. 2022). Transcriptomic changes during PTI signalling are known to heavily overlap with general stress responses, and the majority of PTI responding genes have been observed upregulated also by other types of stresses, including wounding, cold, salt or drought (Björnson et al. 2021). This was also observed in younger *Pc*GCE‐expressing aspen leaves (Table S6) and *A. thaliana* (Tsai et al. 2017) but in older leaves, the abiotic‐stress related genes were largely downregulated (Table S2). Importantly, both aspen and *A. thaliana* expressing *Pc*GCE showed induction of two key PTI response markers which are not responsive to other types of stresses in *A. thaliana* : glutamate receptor *GLR2.7* and transcription factor *NAC61* in younger leaves and genes involved in JA signalling (Table S6) (Björnson et al. 2021). This supports the idea that *Pc*GCE protein induces stress in both species accompanied by activation of surface PRRs.

PRRs are activated by molecular patterns derived either from pathogens (PAMPs/MAMPs) or from native host components (DAMPs). Tsai et al. (2017) suggested two alternative hypotheses of the molecular action of *Pc*GCE; either that *Pc*GCE could induce DAMPs recognised by PRRs or that the *Pc*GCE protein itself is recognised as a PAMP. We found that the mutated *Pc*GCE^S217A^ that was enzymatically inactive (Figure S2) was at least as effective as the active one in inducing premature senescence (Figure 7), excluding the possibility that enzymatically produced DAMPs by glucuronoyl esterase activity induced leaf senescence, leaving the PAMP hypothesis. The latter mechanism assumes that exogenous *Pc*GCE or *Pc*GCE^S217A^ is recognised by a surface PRR perceiving their specific motifs and transducing the signals further, activating ROS and many PTI‐related genes. However, our elicitation attempts failed to show the induction of ROS in aspen or the induction of genes that were highly induced by overexpression of *Pc*GCE (Figure 8). Although not all elicitors rapidly induce ROS (Souza et al. 2017), the lack of any effect on marker genes strongly suggests that *Pc*GCE is not an elicitor recognised as a PAMP.

As *Pc*GCE induced immune defences when expressed from the *35S* promoter but not from the *WP* promoter (Figure 5), it is possible that these effects were dependent on the level of transcript accumulation (Figure 3). High transcript accumulation observed in *35S:PcGCE* expressing plants could lead to oversaturating the protein biosynthesis machinery, leading to ER stress, which induces the unfolded protein response (UPR) (Liu and Howell 2016). The stress is sensed in the ER by the conserved inositol‐requiring enzyme 1 (IRE1) that is a bifunctional kinase and ribonuclease, mediating unconventional splicing of specific TFs such as bZIP60, which transcriptionally regulates UPR target genes. In addition, IRE1 destroys specific mRNAs inhibiting their translation to restore the protein folding ability of the ER (Pastor‐Cantizano et al. 2024). IRE1 has a dual role, maintaining ER homeostasis by activating UPR when the stress is mild and triggering PCD via BAP2 protein when it is acute (Yang et al. 2016; Pastor‐Cantizano et al. 2024). We did find some marker genes that are associated with UPR (Howell 2017) upregulated in leaf 11 but not so much in leaf 8 (Table S6), suggesting that there is a threshold of misfolded protein for the activation of UPR that is not yet reached in Leaf 8, but evident in Leaf 11. Notably, both *IRE1* Arabidopsis homologues, *IRE1A* and *IRE1B*, were upregulated. Moreover, the *BAP2* gene was upregulated, indicating that the ER stress in Leaf 11 progressed enough to trigger PCD. Some other examples include UPR markers *SHD*, *ATERDJ3A*, induced both in aspen and *Arabidopsis* and three homologues of *NF‐YB3* induced in aspen leaf 11.

Exponential accumulation of *PcGCE* transcripts in developing leaves (Figure 3) suggests that it is controlled by a positive feedback loop. Such a loop could operate when *Pc*GCE induces a condition that, in turn, activates *35S* promoter activity. For example, the increase in ROS could stimulate the *35S* promoter by oxidative stress‐induced TGA1 (Rüth et al. 1994). Senescence‐ and abiotic stress‐induced increases in *35S* promoter activity were also reported (Kiselev et al. 2021). The exponential increase of *PcGCE* transcript started in leaf 14 and older, suggesting that a threshold for *PcGCE* transcript level is reached at this leaf developmental stage. No significant stress symptoms were observed when *Pc*GCE was expressed in short‐lived xylem cells, suggesting that there was no sufficient *PcGCE* transcript accumulation during xylogenesis to trigger the avalanche of immune responses. There was, however, a subtle increase in ROS in the leaves of plants expressing *Pc*GCE from the WP promoter (Figure 5), which could indicate slight activation of immune responses.

Thus, although the molecular mechanism of stress symptoms induction by ectopically expressed *Pc*GCE is still unclear, we excluded the DAMP and PAMP hypotheses, leaving the UPR as the most likely alternative mechanism. Whether such a mechanism is involved in other plant responses to transgenes, for example, the reported stress induction by *Aspergillus nidulans* α‐arabinofuranosidase (*An*AF54) (Tsai et al. 2017) remains to be established.

It is promising to find no adverse effects and possibly slightly improved growth when *Pc*GCE is expressed from the *WP* promoter. It remains to be established if such plants have better lignocellulose properties for saccharification, as suggested by previous studies (Tsai et al. 2012; Gandla et al. 2015). We address this point in the accompanying publication (Derba‐Maceluch et al. 2025). It is also of interest to investigate if the low level of ROS induction in *WP:PcGCE* transgenic hybrid aspen lines could lead to better stress resilience by priming effect.

## 4 Experimental Procedures

### 4.1 Generation of Transgenic Aspen Lines

*Phanareochete carnosa* Burt glucuronoyl esterase (*Pc*GCE) cDNA (NCBI accession: JQ972915; Tsai et al. 2012) with its native signal peptide replaced by the signal peptide of *Ptt*Cel9B3 (GenBank accession AY660968.1) was used in the vector *35S:PcGCE* described in the previous publications (Tsai et al. 2012; Gandla et al. 2015). The transgene from this vector was used for the cloning of *WP:PcGCE* construct in the vector pK‐pGT43B‐GW7 (Ratke et al. 2015) containing the *WP* promoter. Point mutation S217A in the *Pc*GCE active site that abolished esterase activity was previously described (Tsai et al. 2012). It was incorporated by PCR using overlapping primers (Table S8) and the chimeric mutated transgene was subsequently subcloned into binary vector pK2WG7.0 (Karimi et al. 2002) using Gateway System (Invitrogen).

Vectors were transferred into competent *Agrobacterium tumefaciens* (Smith and Townsend) Conn strain GV3101 by electroporation and used to transform hybrid aspen, *Populus tremula* L. x *tremuloides* Michx., clone T89 as previously described (Gandla et al. 2015). Twenty independent lines were obtained and clonally propagated, and between two and four lines of each construct with the highest expression levels were selected for analyses.

### 4.2 Plant Cultivation Conditions

The plants were grown in a greenhouse with natural light in Umeå, Sweden (63.8258° N, 20.2630° E) supplemented with illumination from Fiona Lightning 300 (Senmatic, Sonderso, Denmark) to the light intensity of approximately 200 μmol/s/m^2^ and an 18 h photoperiod with a temperature day/night of 20°C/15°C and 60% relative humidity.

### 4.3 Glucuronoyl Esterase Activity

Wall bound proteins were extracted and desalted using a column (Nanosep 30 k omega) as described previously (Gandla et al. 2015). Glucuronoyl esterase activity was measured using benzyl‐D‐glucuronate (Santa Cruz Biotechnology) as a substrate. The reaction having a final volume of 40 μL (50 mM sodium phosphate buffer pH 6.0, 5 mM substrate and 5 μg of protein) was incubated at 30°C for 60 min and the remaining substrate was quantified by Hestrin's method (Hestrin 1949). The standard curve was generated using benzyl‐D‐glucuronate.

### 4.4 Analysis of Calcium Oxalate Crystals

Successive leaves of four plants per genotype were fixed in 70% ethanol and cleared in a 2.5% commercial bleach solution of sodium hypochlorite until the chlorophyll was removed. Sections from the tip, middle and base of each leaf were washed with water, mounted in 50% glycerol and observed using an Axioplan 2 microscope (Zeiss, Germany) and Nomarsky's optics. Counts per vein area were performed using Image J. The chemical nature of crystals was determined by x‐ray diffraction (XRD) using veins from leaf 20 of *35S:PcGCE*‐10 plants and a Bruker D8 advance x‐ray diffractometer with Cu Kα radiation in Ɵ:Ɵ mode, equipped with a super speed VÅNTEC‐1 detector. The samples mounted on a Si low‐background rotating sample holder were analysed by continuous scanning for at least 4 h. Crystals were identified using Bruker software and the powder diffraction file PDF‐2 (International Center for Diffraction Data).

### 4.5 Dye Uptake Experiments

To monitor hydraulic continuity of xylem, small lateral branches of *35S:PcGCE‐10*, *‐4* and WT plants were placed in tubes containing 4% basic fuchsin for 3–4 h and photographed with illumination from above.

### 4.6 DAB Staining

Sections (1 cm^2^) of leaves were incubated in 1 mL of diaminobenzidine tetrahydrochloride (DAB) solution (1 mg/mL, pH 7.0) at room temperature in the dark for 24 h, washed with water, cleared in 95% ethanol at 37°C for 24 h, rehydrated using a graded ethanol series and mounted in 50% aqueous glycerine for observation using Axioplan 2 microscope. The micrographs were converted to grey scale between 0 and 256 using Python's library OpenCV (https://pypi.python.org/pypi/opencv‐python). The area of dark pixels (grey scale 0–31) considered as DAB signals was calculated as % of total area.

### 4.7 Microscopy of Cuticle

Leaf sections of three plants per genotype were fixed in 2.5% glutaraldehyde, embedded in Steedman's wax, stained for lipids with Nile red (Sigma‐Aldrich) according to Dobrowolska et al. (2015) and examined by epifluorescence microscopy (Axioplan 2; Zeiss, Germany) with excitation 450–490 nm and emission above 520 nm. Cuticle thickness was measured using Image J in six sections per tree at six random places per section for abaxial and adaxial cuticle.

### 4.8 Transcriptomics

Developing leaves (L8, L11, L21 and L23) were collected from 10‐week‐old hybrid aspen. RNA was extracted as described by Ratke et al. (2015). Leaves L8 and L11 were analysed in five plants of *35S:PcGCE‐10* and WT. Leaves L21 and L23 were analysed in four plants of *35S:PcGCE‐10*, *23*, *WP:PcGCE‐8*, *‐14* and eight plants of WT. cDNA was sequenced using Illumina HiSeq‐PE150 by Novogene Bioinformatics Technology Co. Ltd. (Beijing). Quality control and mapping to *P. trichocarpa* transcriptome v3.0 were performed by Novogene (L8, L11) or RNA‐Seq raw data were filtered and mapped as described by Kumar et al. (2020) (L21, L23). Raw counts were used for differential expression analyses using DESeq2. *p*‐values were corrected for multiple testing using the Benjamini and Hochberg method to calculate *p*_adj_. Genes were considered as DE when *p*_adj_ < 0.05 and abs(Log_2_FC) > 0.3. R (v3.4.0; https://www.R‐project.org) and Python (Van Rossum and Drake 2009) programmes were used for gene sorting, filtering, intersection, sample grouping and biological function summary.

### 4.9 Metabolomics

Frozen leaf powder (9–12 mg) from L21 to L23 was extracted in 500 μL of extraction buffer [20/20/60 v/v chloroform (Darmstadt, Germany): deionised water (Milli‐Q): methanol (Waltham, MA, USA)] including internal standards (Gullberg et al. 2004): L‐proline‐^13^C5, alpha‐ketoglutarate‐^13^C4, myristic acid‐^13^C3 and cholesterol‐D7 (Andover, MA, USA), as well as succinic acid‐D4, salicylic acid‐D6, L‐glutamic acid‐^13^C5,15 N, putrescine‐D4, hexadecanoic acid‐^13^C4, D‐glucose‐^13^C6, D‐sucrose‐^13^C12 (Sigma, St. Louis, MO, USA) in a bead mixer mill at 30 Hz for 3 min, centrifuged at +4°C, 20 000 *g*, for 10 min and 75 μL of supernatant was transferred to a micro vial and dried. Derivatization was performed according to Gullberg et al. (2004) and 0.5 μL of each sample was injected in splitless mode by a L‐PAL3 autosampler (CTC Analytics AG, Switzerland) into an Agilent 7890B gas chromatograph equipped with a 10 m × 0.18 mm fused silica capillary column with a chemically bonded 0.18 μm Rxi‐5 Sil MS stationary phase (Restek Corporation, USA). The injector temperature was 270°C, the purge flow rate was 20 mL min^−1^ and the purge was turned on after 60 s. The gas flow rate through the column was 1 mL min^−1^, the column temperature was held at 70°C for 2 min, then increased at a rate of 40°C min^−1^ to 320°C and maintained for 2 min. The column effluent was introduced into the ion source of a Pegasus BT time‐of‐flight mass spectrometer, GC/TOFMS (Leco Corp., St Joseph, MI, USA). The transfer line and the ion source temperatures were 250°C and 200°C, respectively. Ions were generated by a 70 eV electron beam at an ionisation current of 2.0 mA, and 30 spectra s^−1^ were recorded in the mass range m/z 50–800. The acceleration voltage was turned on after a solvent delay of 150 s. The detector voltage was 1800–2300 V.

### 4.10 Hormonomics

Samples of L21 and L23 were extracted, purified and analysed according to the method described in Šimura et al. (2018). Mass spectrometry analysis of targeted compounds (Table S9) was performed by an UHPLC‐ESI‐MS/MS system comprising a 1290 Infinity Binary LC System coupled to a 6490 Triple Quad LC/MS System with Jet Stream and Dual Ion Funnel technologies (Agilent Technologies, Santa Clara, CA, USA). The quantification was carried out in Agilent MassHunter Workstation Software Quantitative (Agilent Technologies, Santa Clara, CA, USA).

### 4.11 Fatty Acid Methyl Esters (FAMEs) Analysis

Frozen leaf powder (19–21.6 mg) (L10, L13, L15 of four plants per genotype) was extracted with 500 μL of extraction buffer [2:1 v/v chloroform (Darmstadt, Germany): methanol (Waltham, MA, USA)] following a modified Folch's protocol (Diab et al. 2019). Fatty acids were converted to methyl esters by methylation with diazomethane. For quantification, a calibration curve was prepared from Supelco 37 Component FAME Mix (Sigma‐Aldrich). Analysis was performed by GC‐QqQ‐MS equipped with a Zebron ZB‐FAME 20 m × 0.18 mm internal diameter fused silica capillary column.

### 4.12 Data Analysis /Statistical Methods for Metabolomics

For the GC–MS data, all non‐processed MS files from the metabolic analysis were exported from the ChromaTOF software in NetCDF format to MATLAB R2016a (Mathworks, Natick, MA, USA), where all data pre‐treatment procedures, such as baseline correction, chromatogram alignment, data compression and multivariate curve resolution, were performed using custom scripts. The extracted mass spectra were identified by comparisons of their retention index and mass spectra with libraries of retention time indices and mass spectra 3 using NIST MS 2.0 software. Annotation of mass spectra was based on reverse and forward searches in the library. Principal component analysis (PCA) was performed in the R (v3.4.0; https://www.R‐project.org) programme, by using the ggfortify (https://CRAN.R‐project.org/package=ggfortify) package.

### 4.13 Reverse Transcription‐Quantitative Polymerase Chain Reaction

For hybrid aspen, the RNA was extracted from leaves and developing xylem (Ratke et al. 2015) of three to four plants per genotype. For Arabidopsis seedlings, the RNA was extracted using TRI Reagent TM (Applied Biosystems, Bedford, MA, USA). DNA was removed using (DNA‐free DNA Removal Kit; Thermo Fisher Scientific, Uppsala, Sweden). cDNA was synthesised using Bio‐Rad iScript cDNA synthesis kit. Quantitative polymerase chain (qPCR) reactions were performed using LIGHTCYCLER 480 SYBR GREEN I Master Mix (Roche) and primers (Table S8), either in 20 μL volume [LightCycler 480 System II (Roche)], or in 5 μL volume [C1000 Touch thermal cycler (Bio‐Rad)] and the programme: 95°C for 5 min, then 50 cycles of 95°C for 30 s, 60°C for 15 s and 72°C for 30 s. *UBQ‐L* (*Potri.005G198700*) was selected from four tested genes as the reference for hybrid aspen, based on GeNorm (Vandesompele et al. 2002) analysis and *AtACTIN2* (*AT3G18780*) was used as a reference gene for Arabidopsis. The relative expression level was calculated according to Pfaffl (2001) in Python (Van Rossum and Drake 2009).

### 4.14 Grafting

The scions of 5‐week‐old plants excised 5–10 cm below the shoot apex had their stems trimmed into wedges, inserted into longitudinally cut rootstocks and sealed with Parafilm (Pechiney Plastic Packaging Company). The shoots of the grafted plants were enclosed in plastic bags for 7–10 days. In some plants, all the leaves were removed from the rootstocks on the day of grafting and all side shoots were removed as they appeared.

### 4.15 Purification of PcGCE Expressed in *P. pastoris*

*Pitchia pastoris* (Guillermond) Pfaff strain GS115 expressing mutated *Pc*GCE^S217A^ was obtained as described previously (Tsai et al. 2012). The strains were grown for 24 h in non‐inductive buffered glycerol complex medium [BMGY: 1% yeast extract, 2% peptone, 100 mM potassium phosphate (pH 6.0), 1% yeast nitrogen base with ammonium sulphate (YNB) without amino acids, 4 × 10^−5^% biotin, 1% glycerol], followed by 4 days in inductive buffered methanol complex medium (BMMY) obtained by adding methanol to BMGY to a final concentration of 0.5% and then repeating the additions of the same volume every 24 h during 72 h. The medium containing the recombinant protein was purified by affinity chromatography Ni‐NTA agarose (cOmplete His‐Tag Roche).

### 4.16 Luminol Assay for ROS

Leaf disc was dissected with a biopsy punch (4 mm diameter) from a young, expanded leaf of a 7‐week‐old hybrid aspen, loaded into a well of a 96‐well plate (Thermo Fisher Scientific) containing 100 μL of water and incubated at room temperature in the dark overnight. The water was replaced with 100 μL of assay solution [50 mM phosphate buffer pH 6.5, 50 μM L‐012 (Wako Chemicals), 10 μg/mL peroxidase from horseradish (Type VI‐A, Sigma) and 200 nM of one of the tested elicitors: flg22 (Ezbiolab), purified *Pc*GCE, purified mutated *Pc*GCE^S217A^ or no elicitor (control)]. Light emission was measured every other minute for 40 min using high‐resolution photon counting system (HRPCS5 PHOTEK). The integrated total photon count from each sample was calculated with Image32 (6.0.3, Photek, East Sussex, UK), and the results from five biological replicates, each based on 16 technical replicates, obtained after 20 min elicitation time, were displayed. The experiment was repeated three times with similar results.

### 4.17 Induction of Marker Genes by *Pc*GCE in Arabidopsis

Seeds of *Arabidopsis thaliana* ecotype Columbia (Col‐0) were surface sterilised with ethanol and subsequently grown in MS medium supplemented with 0.5% sucrose, in 12‐well plates (20–25 seeds/plate). After 9 d in long‐day conditions (16 h light/8 h dark), the seedlings were treated with elicitors by replacing the growth medium with the medium containing 1 μM of either: flg22 (Ezbiolab), purified *Pc*GCE, purified mutated *Pc*GCE^S217A^, bovine serum albumin (BSA) or no elicitor (buffer). The seedlings were frozen in liquid N_2_ after 1 h and 3 h of treatment and used for RNA isolation and RT‐qPCR.

### 4.18 Univariate Statistical Analyses

For all univariate statistical analyses, we used ANOVA, followed by one of the three post hoc tests. The Dunnett's test was used when two or more means were compared to a control; a *t*‐test was used when two means were compared, and Tukey's test was used for multiple comparisons of several means.

## Author Contributions

E.N.D. performed the majority of experiments (gene expression, transcriptomics, metabolomics, grafting, phenotyping, elicitor activity testing) and wrote the manuscript; M.D.‐M. created transgenic aspen lines and supervised phenotyping and vector cloning; X.‐K.L. performed initial phenotyping; H.C.B. performed RT‐qPCR analyses; I.D. performed microscopy of leaves; M.T. and D.B. analysed crystals; J.L. analysed tyloses and gels and performed dye uptake experiments; J.Š. and K.L. performed hormonomics; L.A.K. supervised enzymatic analyses; M.E.E. supervised the luminol assay; A.Y.‐L.T. and E.R.M. cloned and expressed *Pc*GCE in *Pichia* and designed the active site mutation; K.S.K. tested different methodologies for the elicitor activity; E.J.M. designed and coordinated the study and finalised the manuscript.

## Conflicts of Interest

The authors declare no conflicts of interest.

## Supporting information


**Figure S1:** Principal component analysis of free fatty acid contents of leaves 10, 13 and 15 in transgenic (*35S:PcGCE‐10*) and WT plants showing separation of expanding (L10) and expanded (L13 and L15) leaf samples.

**Figure S2:** Specific activity of *Pc*CGE and mutated *Pc*GCE^S217A^ expressed in *Pichia pastoris*.

**Table S1:** All differentially expressed genes in each transgenic line expressing *PcGCE*, *35S:PcGCE‐10* and *‐23*, and each leaf developmental stage, leaf 21 and leaf 23, sorted according to MAPMAN biological process.
**Table S2:** Differentially expressed genes in both transgenic lines expressing *Pc*GCE, *35S:PcGCE*‐*10* and *‐23*, and either leaf developmental stage, leaf 21 and leaf 23, listed according to functional classification.
**Table S3:** All differentially expressed genes in each transgenic *WP:PcGCE* line, *WP:PcGCE‐ 8* and *‐14* and each leaf developmental stage, leaf 21 and leaf 23, sorted according to MAPMAN biological process.
**Table S4:** Differentially abundant hormones, their degradation products and their precursors in leaves 21 and 23 of transgenic plants *(WP:PcGCE‐8* and *‐14*, combined) compared to WT.
**Table S5:** Differentially abundant metabolites in leaves 21 and 23 of transgenic plants (*WP:PcGCE‐8* and *‐14*, combined) compared to WT.
**Table S6:** All differentially expressed genes in transgenic line *PcGCE‐10* at two developmental stages, leaf 8 and leaf 11, sorted according to MAPMAN biological process.
**Table S7:** Gene ontology (GO) enrichment analysis of DE genes in leaf 8 and 11 of *35S:PcGCE‐10* plants compared to WT.
**Table S8:** Primers used in this study.
**Table S9:** Phytohormones and related compounds quantified by hormonomics analysis.
**Table S10:** Codes of samples deposited at the European Nucleotide Archive (ENA) at EMBL‐EBI (https://www.ebi.ac.uk/ena/browser/home), under accession ID PRJEB47981.

## Data Availability

The data that support the findings of this study are openly available in European Nucleotide Archive (ENA) at EMBL‐EBI at https://www.ebi.ac.uk/ena/browser/home, reference number PRJEB47981.
