# Supplementary material for: Glucuronoyl Esterase of Pathogenic Phanerochaete carnosa Induces Immune Responses in Aspen Independently of Its Enzymatic Activity
Source: Plant Biotechnol J. 2025 Sep 17;24(2):602–19. doi: 10.1111/pbi.70357 (PMC12906814; doi:10.1111/pbi.70357)
Supplement: Supplementary file 1 — Figure S1: Principal component analysis of free fatty acid contents of leaves 10, 13 and 15 in transgenic (35S:PcGCE‐10) and WT plants showing separation of expanding (L10) and expanded (L13 and L15) leaf samples. [file PBI-24-602-s002.pdf]

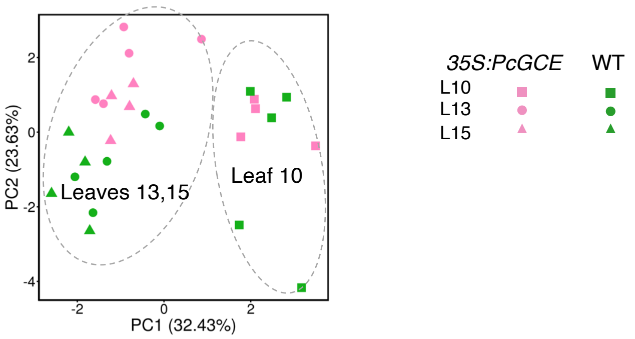

Figure S1. Principal component analysis of free fatty acid contents of leaves 10, 13, and 15 in transgenic (*35S:PcGCE-10*) and WT plants showing separation of expanding (L10) and expanded (L13 and L15) leaf samples.
