# Supplementary material for: Glucuronoyl Esterase of Pathogenic Phanerochaete carnosa Induces Immune Responses in Aspen Independently of Its Enzymatic Activity
Source: Plant Biotechnol J. 2025 Sep 17;24(2):602–19. doi: 10.1111/pbi.70357 (PMC12906814; doi:10.1111/pbi.70357)
Supplement: Supplementary file 2 — Figure S2: Specific activity of PcCGE and mutated PcGCES217A expressed in Pichia pastoris. [file PBI-24-602-s003.pdf]

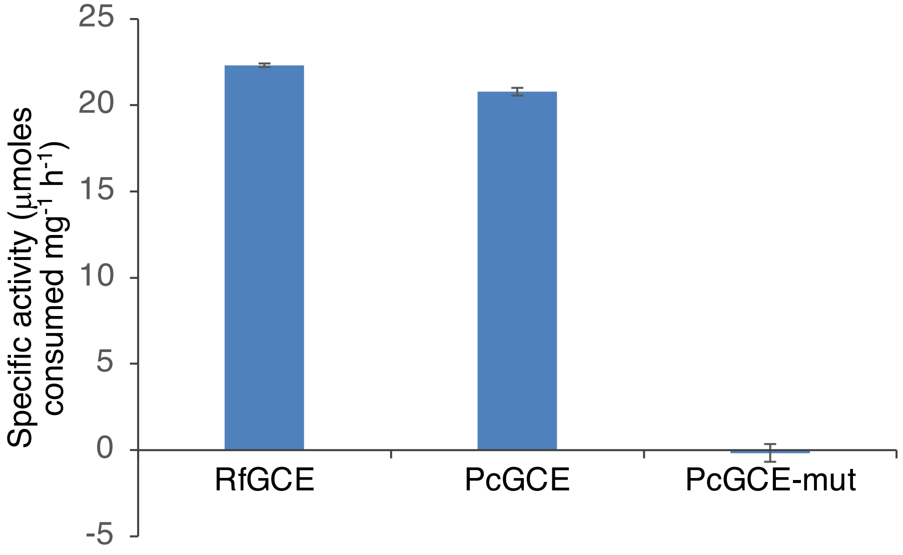

Figure S2. Specific activity of *Pc*CGE and mutated S217A *Pc*GCE^{S217A} expressed in *Pichia pastoris*. *Ruminococcus flavefaciens* glucuronoyl esterase (*Rf*GCE) from Megazymes was used as positive control. Data are means ± SE, N = 3.
